# Global Optimal Solving Algorithm for Power Distribution Based on Selective Harmonic Elimination in Cascaded H-Bridge Multilevel Inverters

**DOI:** 10.3390/s25113524

**Published:** 2025-06-03

**Authors:** Xingyue Qian, Jun Hao, Jiajia Xiao, Hanzhi Yang, Qi Zhang, Zhibao Yuan

**Affiliations:** 1 College of Automation, Jiangsu University of Science and Technology, Zhenjiang 212100, China; xingyueqian@stu.just.edu.cn (X.Q.); 232210301218@stu.just.edu.cn (J.X.); 232210305205@stu.just.edu.cn (H.Y.); 2 School of Artificial Intelligence, China University of Mining and Technology (Beijing), Beijing 100083, China; qz@cumtb.edu.cn; 3 Key Laboratory of High-Density Electromagnetic Power and Systems (Chinese Academy of Sciences), Institute of Electrical Engineering, Chinese Academy of Sciences, Beijing 100190, China; yuanzhibao@mail.iee.ac.cn

**Keywords:** selective harmonic elimination, power distribution, polynomial homotopy continuation, cascaded H-bridge inverters, global optimal solutions

## Abstract

The conventional power distributions methods face many challenges, such as high switching frequency, multiple carrier cycles, and single power distribution. To address the above issues, a novel power distribution based on the selective harmonic elimination (PD–SHE) strategy is proposed to achieve arbitrary power distribution and selective harmonic elimination but with low switching frequency and single carrier cycle. Firstly, the novel PD–SHE model is established based on the principles of SHE and power calculation theory, where distribution ratio is introduced to adjust power distribution arbitrarily and its constraints have been deduced. In addition, the issue of redundancy of solution is also analyzed and solved by adding valid constraints. Finally, polynomial homotopy continuation (PHC) algorithm is applied to solve the novel PD–SHE model. Then, all the physically realizable solutions can be found without choosing the initial value in the full range of modulation index. The results of simulation analysis show the effectiveness of PD–SHE strategy and the superiority of PHC algorithm in solving the global optimal solution. Moreover, the reliability of the global optimal solution for PD–SHE strategy is verified via physical experiments in terms of harmonic elimination and active power distribution.

## 1. Introduction

There are numerous advantages of selective harmonic elimination (SHE) technology. For examples, the selective low-frequency harmonics can be precisely eliminated while maintaining a low switching frequency and low switching losses [1,2,3,4,5]. Thus, the performance of the system and the quality of the output waveform can be improved. Therefore, it is commonly employed in medium-power and high-power applications [6], especially in the field of multilevel converters [7].

In recent years, SHE has been combined with other technologies by global researchers, creating new methods. For instance, model predictive control (MPC) and SHE are combined to balance the capacitor voltages well and retain the advantages of SHE in hybrid-clamped (HC) inverters [8]. In [9], MPC and SHE are integrated to produce an optimized voltage waveform without undesired commutations and oscillations. In addition, the capacitor charge objective is integrated with the nonlinear transcendental equations of SHE to solve the imbalance problems of DC-link capacitors and flying capacitors (FC) in four-level neutral-point-clamped (NPC) converters. Hence, the capacitor voltage can be balanced naturally [10]. Furthermore, a method is proposed to apply SHE in parallel three-phase inverters which suppresses zero-sequence circulating currents and eliminate the harmonics [11]. In the case of the same switching frequency, its efficiency in reducing total harmonic distortion (THD) is better than other methods.

In the application process of cascaded multilevel inverters using SHEPWM, the power distributions among cascaded modules have obvious flaws due to the differences in switching angles, that is, some modules have a high output power, while others have a low output power. Such an issue would directly affect the lifespans of the modules and even cause serious safety accidents. By combining SHEPWM with power calculation theory, the power balance based on the SHE (PB–SHE) strategy is designed. This strategy can ensure that selective low-frequency harmonics are eliminated and the power among cascaded modules are balance. This has important practical significance for extending the lifespans of power electronic equipment and avoiding system efficiency reduction or even major safety accidents caused by imbalance. Although robust multicarrier modulation (RMM) is proposed in [12] to achieve power sharing under equal DC voltage, a PB–SHE strategy has lower switching losses than RMM. A carrier-shifting strategy synchronized with the modulation period is proposed in [13] to balance power in cascaded H-bridge units. However, it suffers from slower balancing with more units, whereas the PB–SHE strategy avoids this issue. Moreover, the PB–SHE technique is distinguished by its ability to precisely eliminate targeted harmonics (e.g., 5th, 7th, 11th, 13th) through optimized switching angles, significantly improving output voltage waveform quality and reducing harmonic distortion.

Therefore, it can be seen that PB–SHE has many advantages over conventional SHEPWM technology. However, PB–SHE strategy cannot achieve the arbitrary power distribution of cascaded modules in industrial applications. Moreover, the issue of the global optimal solution for PB–SHE equations has not been solved either.

To address the aforementioned issues, a novel power distribution strategy based on SHE (PD–SHE) strategy is first proposed in this article to achieve arbitrary adjustment of module power distribution. Additionally, the global optimal solutions of PD–SHE equations are also solved by PHC algorithm. The main contributions and innovations of this article can be summarized as follows.

(1) The PD–SHE equations are first proposed. The distribution ratio is introduced to achieve arbitrary adjustment of module power distribution, and the upper and lower bounds of distribution ratio are analyzed and deduced. The reasons for redundancy of solutions in PD–SHE equations are theoretically analyzed.

(2) Technically, some valid constraints are added to effectively avoid the redundancy of the solution. Moreover, the PHC algorithm as an algebraic algorithm is adopted to solve the PD–SHE equations. All the physically possible solutions can be found without choosing initial values for each modulation index. If no solutions can be found for certain a modulation index by PHC algorithm, it guarantees a fact that there are no theoretical solutions for such modulation index.

(3) Compared with conventional power distributions methods, PD–SHE can achieve arbitrary power distribution and selective harmonic elimination but with low switching frequency and single carrier cycle.

The remainder of this article is organized as follows: The PD–SHE equations for typical 5-levels inverter is constructed, and the establishment process of PD–SHE equations is carefully deduced in Section 2. In Section 3, PHC algorithm is introduced in detail. In Section 4, the PHC algorithm is utilized to obtain all of the possible theoretical solutions of PD–SHE equations, and then the global optimal solutions with minimal THD are obtained. In Section 5, two popular intelligent algorithms are carried out to demonstrate the superiority of the PHC algorithm for the global optimal solutions. Physical experiments are presented to verify the reliability of the global optimal solutions solved by the PHC algorithm in Section 6. Finally, this article is concluded in Section 7.

## 2. The Introduce of Noval PD–SHE Strategy

The PD–SHE strategy is introduced in this section. The cascaded H-bridge 5-levels inverter is a typical example to exhibit the establishment process of PD–SHE equations.

**Figure 1:**
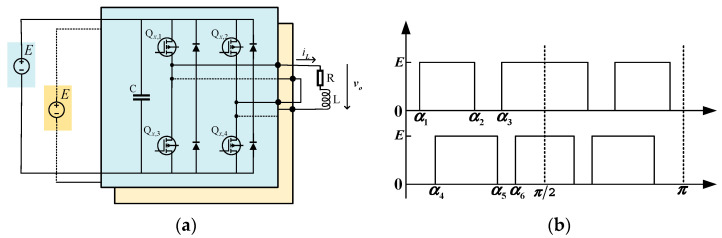
A single-phase H-bridge 5-levels inverter. (**a**) The typical structure of circuit. (**b**) The switching pattern of the switching angles.

Firstly, the active power formulation of each module is derived. In Figure 1a, $v_{o}$ is the output voltage of two cascaded H-bridge inverters. $i_{L}$ is the current of load and the output current of each module. $v_{f}$ is the fundamental voltage of $v_{o}$. The switching pattern shown in Figure 1b is selected as an example to construct equations. The phase angle of $i_{L}$ lagging behind $v_{f}$ is ϕ. Thus, $v_{f}$ and $i_{L}$ can be expressed as

(1) $$v_{f}=V_{f}\sin\omega t,$$

(2) $$i_{L}=I_{L}\sin(\omega t-\phi ),$$

where $V_{f}$ is the amplitude of fundamental output voltage, ω is the angular frequency of $v_{f}$, and $I_{L}$ is the amplitude of the current of the load.

According to [14,15], the real-time output power of the first H-bridge module can be expressed as

(3) $$\begin{array}{cc} p_{o,1} & =v_{f,1}i_{L}\\ & =0.5V_{f,1}I_{L}\cos\phi -0.5V_{f,1}I_{L}\cos(2\omega t-\phi ), \end{array}$$

and the active power of the first H-bridge module can be written as

(4) $$\begin{array}{cc} P_{1} & =0.5V_{f,1}I_{L}\cos\phi \\ V_{f,1} & =\frac{4E}{\pi }(\cos\alpha_{1}-\cos\alpha_{2}+\cos\alpha_{3}) \end{array}$$

Similarly, the active power of the second H-bridge module can be written as

(5) $$\begin{array}{cc} P_{2} & =0.5V_{f,2}I_{L}\cos\phi \\ V_{f,2} & =\frac{4E}{\pi }(\cos\alpha_{4}-\cos\alpha_{5}+\cos\alpha_{6}) \end{array}$$

where $V_{f,1}$ and $V_{f,2}$ are the amplitudes of fundamental voltages of two modules and E is the DC-link voltage. $I_{L}$, and cosϕ are the same with each cascaded module.

According to (4) and (5), it is obvious that the ratio of the output power between modules is identical to the fundamental voltage amplitude of each module. Based on [16], the SHE equations with the switching pattern shown in Figure 1b for cascaded H-bridge 5-levels inverter can be written as

(6) $$\left\{\begin{array}{c} \cos\alpha_{1}-\cos\alpha_{2}+\cdots +\cos\alpha_{6}=2M\\ \sum_{z=1}^{3}{\left(-1\right)}^{z+1}\cos(n\alpha_{z})+\sum_{z=4}^{6}{\left(-1\right)}^{z}\cos(n\alpha_{z})=0, \end{array}\right.$$

with

(7) $$0{}^{\circ}<\alpha_{1}<\alpha_{2}<\alpha_{3}<90{}^{\circ},\;0{}^{\circ}<\alpha_{4}<\alpha_{5}<\alpha_{6}<90{}^{\circ},$$

where n is the order of harmonic to be eliminated (e.g., 5th, 7th, 11th, 13th, 17th) and M is the modulation index which can be defined as

$$M=\frac{\pi V_{f}}{8E}.$$

The process of establishing the novel PD–SHE equations is deduced based on (6) and (7).

**Figure 2:**
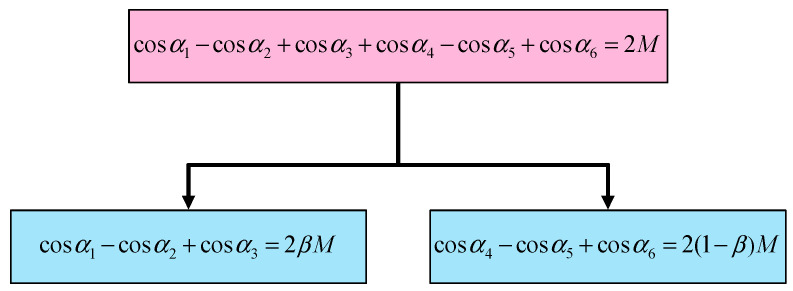
The relationship between SHE and PD–SHE equations.

The first equation in (6) can be split into two equations which determine their respective fundamental voltages. The relationship between SHE and PD–SHE is shown in Figure 2. The parameter β is first introduced to identify the ratio of power distribution between two modules.

**Figure 3:**
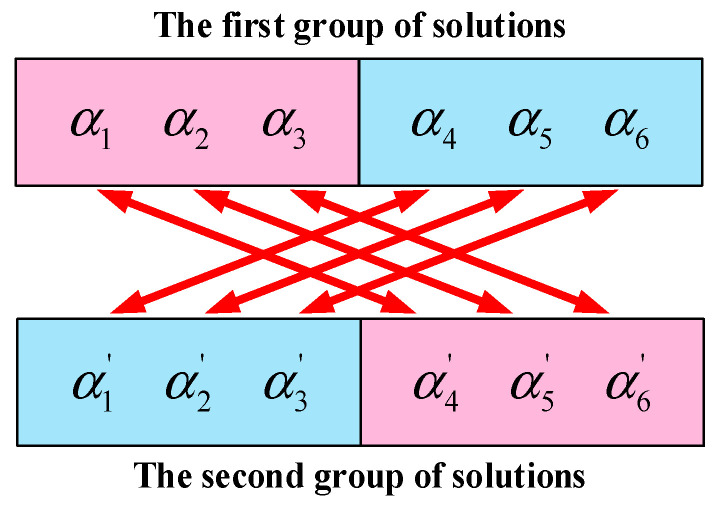
The formation mechanism for solution redundancy.

Since there are multiple groups of solutions of the conventional PB–SHE equations, so as to the redundancy of solution always exists. According to Figure 1, the two modules have essentially no difference between them, which means there is a lack of sequence to distinguish the position of the modules. In other words, $\alpha_{1}$, $\alpha_{2}$, $\alpha_{3}$ in the first module and $\alpha_{4}$, $\alpha_{5}$, $\alpha_{6}$ in the second module can be interchanged. Their substitution will generate two solutions, but they are essentially the same. The diagram of formation mechanism for solution redundancy can be expressed as Figure 3.

Considering the formation mechanism for solution redundancy, PD–SHE equations achieve the goal of distinguishing the order of modules by adding extra constraints. Based on the above strategy, the PD–SHE equations are designed, which is expressed as

(8) $$\left\{\begin{array}{c} \begin{array}{c} \cos\alpha_{1}-\cos\alpha_{2}+\cos\alpha_{3}=2\beta M\\ \;\;\;\cos\alpha_{4}-\cos\alpha_{5}+\cos\alpha_{6}=2(1-\beta )M \end{array}\\ \sum_{z=1}^{3}{\left(-1\right)}^{z+1}\cos(n\alpha_{z})+\sum_{z=4}^{6}{\left(-1\right)}^{z}\cos(n\alpha_{z})=0 \end{array}\right.,$$

with

(9) $$0{}^{\circ}<\alpha_{1}<\alpha_{2}<\alpha_{3}<90{}^{\circ},\;0{}^{\circ}<\alpha_{4}<\alpha_{5}<\alpha_{6}<90{}^{\circ},\;\alpha_{1}<\alpha_{4},$$

where the parameter β is the distribution ratio which determines the power distribution of each module.

Next, the upper and lower bounds of distribution ratio β is derived. Firstly, according to (8), each module needs to have actual active power, one can easily obtain

(10) 0<β<1,

Considering the relationship between the output power in the AC side and the input power in the DC side, it is obvious that M and β should also satisfy the following (11):

(11) $$\left\{\begin{array}{c} 0<2M\beta <1\\ 0<2M(1-\beta )<1 \end{array}\right..$$

According to the inequality principle, (11) can be simplified as

(12) $$\left\{\begin{array}{c} 0<\beta <\frac{0.5}{M}\\ 1-\frac{0.5}{M}<\beta <1 \end{array}\right.’$$

Both lower bound $1-\frac{0.5}{M}$ and upper bound $\frac{0.5}{M}$ are related to the value range of M; therefore, the condition of distribution ratio β can be obtained as (13),

(13) $$\left\{\begin{array}{c} 0<\beta <1,\;0<M<0.5\\ 1-\frac{0.5}{M}\leq \beta \leq \frac{0.5}{M},\;0.5\leq M<1 \end{array}\right..$$

According to (13), the power output of each module can be determined by distribution ratio β and M.

**Remark** **1.** *From the process of derivation, the conventional PB–SHE can be seen as a typical example of PD–SHE model with distribution ratio of 0.5*.

## 3. Global Optimal Solution for the Novel PD–SHE Equations

In this section, the PHC algorithm is adopted to solve the global optimal solutions of the PD–SHE equations [16]. In contrast to common algorithms [17,18,19,20], the PHC algorithm can find the global optimal solutions without initial values and avoid solving failures. The process of how to apply the PHC algorithm to solve PD–SHE is as follows.

According to (8), one can get

(14) $$\left\{\begin{array}{c} \;\;\cos\alpha_{1}-\cos\alpha_{2}+\cos\alpha_{3}-2\beta M=0\\ \cos\alpha_{4}-\cos\alpha_{5}+\cos\alpha_{6}-2(1-\beta )M=0\\ \begin{array}{l} \cos5\alpha_{1}-\cos5\alpha_{2}+\cos5\alpha_{3}+\cos5\alpha_{4}-\cos5\alpha_{5}+\cos5\alpha_{6}=0\\ \cos7\alpha_{1}-\cos7\alpha_{2}+\cos7\alpha_{3}+\cos7\alpha_{4}-\cos7\alpha_{5}+\cos7\alpha_{6}=0\\ \cos11\alpha_{1}-\cos11\alpha_{2}+\cos11\alpha_{3}+\cos11\alpha_{4}-\cos11\alpha_{5}+\cos11\alpha_{6}=0\\ \cos13\alpha_{1}-\cos13\alpha_{2}+\cos13\alpha_{3}+\cos13\alpha_{4}-\cos13\alpha_{5}+\cos13\alpha_{6}=0 \end{array} \end{array}\right.,$$

according to multi-angle formula, such as $\cos(5\alpha )=16\cos^{5}\alpha -20\cos^{3}\alpha +5\cos\alpha$, (14) is rewritten as

(15) $$\left\{\begin{array}{c} \cos\alpha_{1}-\cos\alpha_{2}+\cos\alpha_{3}=2\beta M\\ \;\;\;\cos\alpha_{4}-\cos\alpha_{5}+\cos\alpha_{6}=2(1-\beta )M\\ \begin{array}{l} \sum_{z=1}^{3}{\left(-1\right)}^{z+1}(16\cos{\alpha_{z}}^{5}-20\cos{\alpha_{z}}^{3}+5\cos\alpha_{z})\\ +\sum_{z=4}^{6}{\left(-1\right)}^{z}(16\cos{\alpha_{z}}^{5}-20\cos{\alpha_{z}}^{3}+5\cos\alpha_{z})=0\\ \sum_{z=1}^{3}{\left(-1\right)}^{z+1}(64\cos{\alpha_{z}}^{7}-\cdots -7\cos\alpha_{z})\\ +\sum_{z=4}^{6}{\left(-1\right)}^{z}(64\cos{\alpha_{z}}^{7}-\cdots -7\cos\alpha_{z})=0\\ \sum_{z=1}^{3}{\left(-1\right)}^{z+1}(1024\cos{\alpha_{z}}^{11}-\cdots -11\cos\alpha_{z})\\ +\sum_{z=4}^{6}{\left(-1\right)}^{z}(1024\cos{\alpha_{z}}^{11}-\cdots -11\cos\alpha_{z})=0\\ \sum_{z=1}^{3}{\left(-1\right)}^{z+1}(4096\cos{\alpha_{z}}^{13}-\cdots +13\cos\alpha_{z})\\ +\sum_{z=4}^{6}{\left(-1\right)}^{z}(4096\cos{\alpha_{z}}^{13}-\cdots +13\cos\alpha_{z})=0, \end{array} \end{array}\right.$$

To further simplify Formula (15), let $\cos\alpha_{1}=x_{1}$, $\cos\alpha_{2}=x_{2}$, $\cos\alpha_{3}=x_{3}$, $\cos\alpha_{4}=x_{4}$, $\cos\alpha_{5}=x_{5}$, $\cos\alpha_{6}=x_{6}$. Thus, with the monotonicity of function $\cos\left(\cdot \right)$, (15) can be rewritten as

(16) $$\left\{\begin{array}{c} \;\;\;x_{1}-x_{2}+x_{3}=2\beta M\\ \;\;\;x_{4}-x_{5}+x_{6}=2(1-\beta )M\\ \begin{array}{l} \sum_{z=1}^{3}{\left(-1\right)}^{z+1}(16{x_{z}}^{5}-20{x_{z}}^{3}+5x_{z})\\ +\sum_{z=4}^{6}{\left(-1\right)}^{z}(16{x_{z}}^{5}-20{x_{z}}^{3}+5x_{z})=0\\ \sum_{z=1}^{3}{\left(-1\right)}^{z+1}(64{x_{z}}^{7}-\cdots -7x_{z})\\ +\sum_{z=4}^{6}{\left(-1\right)}^{z}(64{x_{z}}^{7}-\cdots -7x_{z})=0\\ \sum_{z=1}^{3}{\left(-1\right)}^{z+1}(1024{x_{z}}^{11}-\cdots -11x_{z})\\ +\sum_{z=4}^{6}{\left(-1\right)}^{z}(1024{x_{z}}^{11}-\cdots -11x_{z})=0\\ \sum_{z=1}^{3}{\left(-1\right)}^{z+1}(4096{x_{z}}^{13}-\cdots +13x_{z})\\ +\sum_{z=4}^{6}{\left(-1\right)}^{z}(4096{x_{z}}^{13}-\cdots +13x_{z})=0, \end{array} \end{array}\right.$$

with

$$1>x_{1}>x_{2}>x_{3}>0,\;1>x_{4}>x_{5}>x_{6}>0,\;x_{1}>x_{4}.$$

Finally, the PD–SHE equations have been transformed into polynomial Equation (16). For the convenience of representation, (16) can be expressed as (17),

(17) $$\left\{\begin{array}{c} p_{1}(x_{1},x_{2},\cdots ,x_{6})=0\\ p_{2}(x_{1},x_{2},\cdots ,x_{6})=0\\ \vdots \\ p_{m}(x_{1},x_{2},\cdots ,x_{6})=0 \end{array}\right..$$

Homotopy mapping H(x,t) is to be constructed, which can be typically expressed as

(18) H(x,t)=r(1−t)G(x)+tP(x)=0,

where G(x) represents a starting polynomial equation. It is typically chosen for its simplicity. There are three fundamental requirements to be fulfilled when selecting a proper G(x): triviality, smoothness, and accessibility. P(x) is the equation to be solved. r is a random complex number. The parameter t varies from 0 to 1, providing a continuous transition between two equations. When t=0, the homotopy reduces to G(x)=0 and solutions are readily available. When t=1, it transforms to P(x)=0, which represents the equations to be solved in this article. The numerical continuation methods are applied to trace the solution paths defined by the homotopy. Starting from the known solutions at t=0, the value of t incrementally increases as the algorithm tracks the solutions x(t) along their paths.

**Remark** **2.** *According to the ratio theorem of Fabry and Pade approximants, the singularities can be avoided and computed in PHC algorithm. All theoretical solutions without choosing initial values can be found and the descriptions of global convergence are presented in* [21,22].

**Remark** **3.** *PHC software includes all essential components, such as construction of the start system, the homotopy mapping equation, the numerical continuation and path-following algorithms*.

## 4. Results for the Novel PD–SHE Equations

PD–SHE strategy can achieve arbitrary power distribution. Here, distribution ratios 0.5 and 0.6 are taken as typical cases to illustrate.

**Figure 4:**
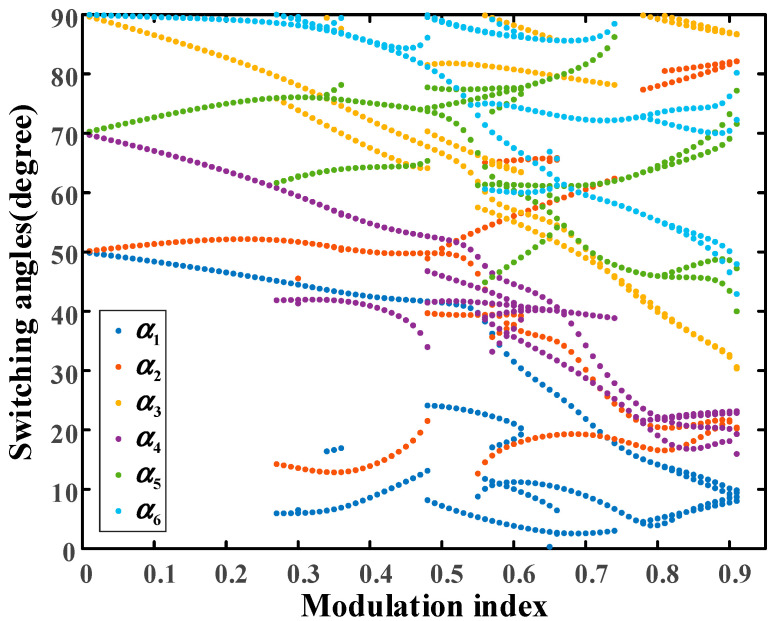
The trajectories of all the solutions versus the full range of modulation index at distribution ratio 0.5.

The case of distribution ratio 0.5 is discussed firstly. The trajectories of all the physically realizable solutions in the full range of the modulation index are shown in Figure 4.

In Figure 4, the range of the modulation index is from 0.01 to 0.91 and the PD–SHE equations are theoretically unsolvable when the modulation index exceeds 0.91. Moreover, it can be clearly seen that there are multiple groups of solutions for some modulation indexes from Figure 4. Due to the fact that there are multiple groups of candidate solutions for some modulation indexes, THD expressed as (20) is selected as the evaluation index to obtain the global optimal solutions among the solution set.

(19) $$THD=\frac{\sqrt{\sum_{m=5,7,11,\cdots }^{49}{V_{m}}^{2}}}{V_{f}}$$

**Figure 5:**
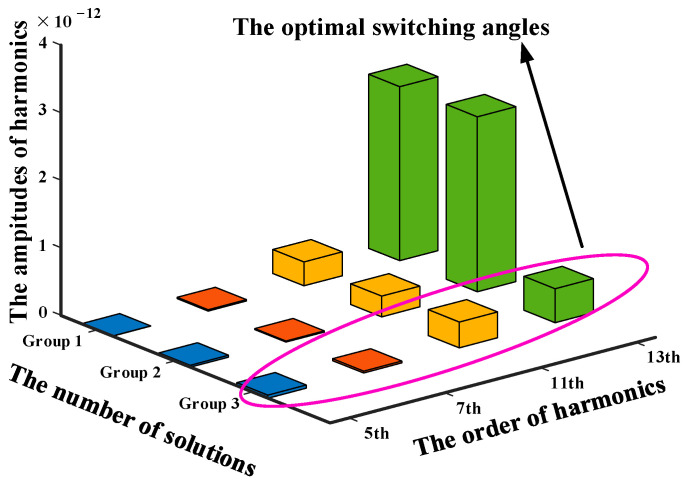
Amplitudes of 5th, 7th, 11th and 13th harmonics for all three groups of solutions solved by the PHC algorithm.

A typical example at distribution ratio 0.5 with modulation index 0.8 is taken to illustrate the selection process of the global optimal solutions. Firstly, there are only three groups of solutions which satisfy the PD–SHE equations. According to (19), the THD of these three candidate groups of solutions can be calculated. Among them, the one that has the minimal THD is determined to be global optimum. The relevant results which are obtained through simulation and calculation are listed in Table 1 and shown in Figure 5.

**Figure 6:**
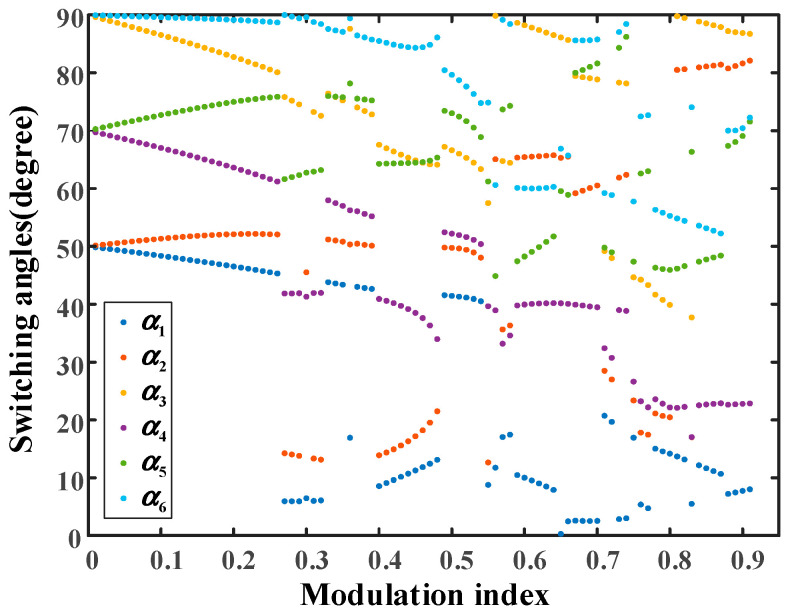
The trajectories of the global optimal solutions versus the full range of modulation index at distribution 0.5.

According to Table 1 and Figure 5, group 3 is the global optimal theoretical solution at modulation index 0.8 and distribution 0.5. The same operations can be conducted for other modulation indexes and the trajectories of the global optimal solutions with minimal THD in the full range of modulation index are shown in Figure 6.

According to Table 1 and Figure 5, group 3 is the global optimal theoretical solution at distribution ratio 0.5 with modulation index 0.8. Similarly, the optimal theoretical solutions with minimal THD for each modulation index have been selected by using (19). Therefore, there is only one group of global optimal theoretical solution for each modulation index. The trajectories of the global optimal solutions in the full range of modulation index are then shown in Figure 6.

To further illustrate that active power between modules can be distributed arbitrarily, the case of distribution ratio 0.6 is also discussed in this section. The global optimal solution at modulation index 0.8 is solved shown in (20). Moreover, the solving processes of other modulation indexes are the same as modulation index 0.8.

(20) $$\begin{array}{ccc} \alpha_{1}=4.81367^{{}^{\circ}} & \alpha_{2}=79.0750^{{}^{\circ}} & \alpha_{3}=81.1963^{{}^{\circ}}\\ \alpha_{4}=38.1153^{{}^{\circ}} & \alpha_{5}=64.6167^{{}^{\circ}} & \alpha_{6}=73.6262^{{}^{\circ}} \end{array}$$

## 5. Comparison and Simulation Verification

In order to demonstrate the superiority of the global optimal solutions solved by the PHC algorithm, the modified particle swarm optimization (MPSO) algorithm [17] and composite differential evolutions (CoDE) algorithm [20] are carried out to solve the PD–SHE equations. The parameters of MPSO algorithm are set as follows: The population size is 50 and the number of iterations is 300. The social $c_{2}$ parameter is constrained within the range of [0.1, 2]. Other parameters are the same as [17]. The parameters of the CoDE algorithm are set as follows: the population size $N_{p}$ is 30 and the number of iterations is 300. The scaling factor F is 1.0 and the cross-control factor $C_{r}$ is 0.9. Other parameters are the same as [20]. Two algorithms are carried out 10 times and the median solution is selected as the final results. The solutions of the PD–SHE equations at modulation index 0.8 with different distribution ratios of these three algorithms are shown in Table 2.

**Figure 7:**
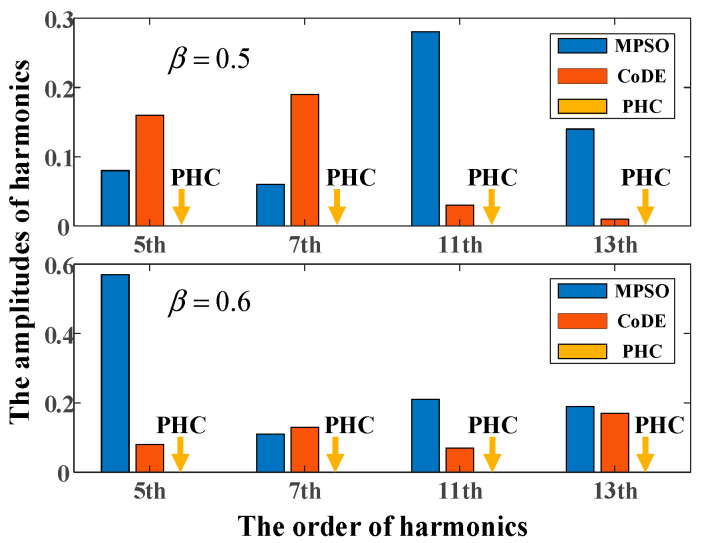
The amplitudes of 5th, 7th, 11th, and 13th harmonics at modulation index 0.8 for three algorithms.

Since different solutions have different modulation abilities for the fundamental voltage, which leads to different denominators for THD. To avoid this negative effect, the total selective harmonic distortion (TSHD) is introduced to evaluate these three groups of solutions and is defined as

(21) $$TSHD=\sqrt{\sum_{i=5}^{13}{V_{i}}^{2}},\;i=5,7,11,13$$

where $V_{i}$ is the amplitude of the ith harmonic. In order to compare these three algorithms in terms of TSHD, the amplitudes of 5th, 7th, 11th, and 13th harmonics are shown in Figure 7 and the values of TSHD are listed in Table 2. From Figure 7, it can be clearly observed that the group of solutions obtained by the PHC algorithm presents the best ability of harmonic elimination.

**Figure 8:**
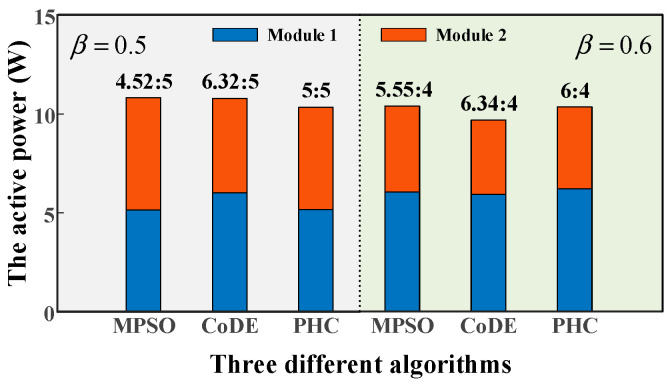
The active power of each module at modulation index 0.8 for three algorithms.

To evaluate the ability of power distribution for these three algorithms, the absolute error of active power (AEAP) is also introduced and is defined as follows:

(22) $$AEAP=\sum_{i=1}^{2}\sqrt{{\left(P_{i}-P_{i}^{*}\right)}^{2}},\;i=1,2$$

where $P_{i}$ is the active power of the ith module and $P_{i}^{*}$ is the theoretical active power of the ith module. In order to compare these three algorithms in terms of AEAP, the active power ratios between modules are shown in Figure 8 and the values of AEAP are listed in Table 2.

As shown in Figure 8, irrespective of what the distribution ratio is, the solutions obtained by the PHC algorithm achieve power distribution completely. However, MPSO and CoDE algorithms may hardly distribute power according to distribution ratio between modules. Based on the previous analysis, the PHC algorithm performs better than the other two algorithms in both selective harmonic elimination and power distribution for cascaded H-bridge 5-level inverter.

## 6. Experiment Validation

In this section, some typical cases for different distribution ratios are carried out to verify the correctness and superiority of the global optimal solutions for the PD–SHE. The theoretical and experimental values of different cases are provided to validate the reliability of the global optimal solutions further. The parameters of the experimental platform are listed in Table 3.

**Figure 9:**
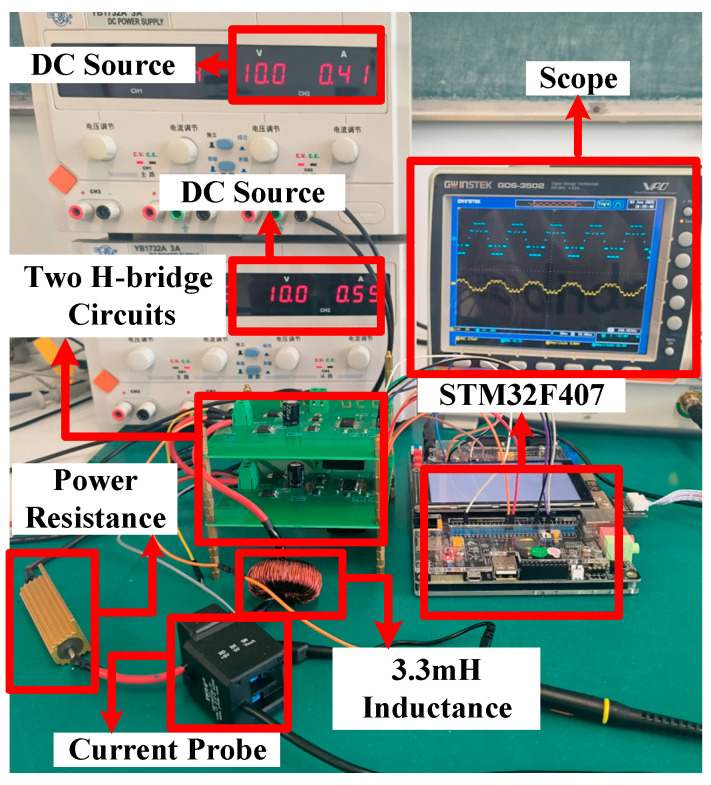
Diagram of the experimental platform.

The main circuit is shown in Figure 9. The FDD8424H (40 V/20 A) is employed as the switching device. The ADuM1400 serves as the isolator, and the FAN3278 functions as the gate driver. An STM32F407 microcontroller which is based on the ARM Cortex-M4 architecture is employed to generate the PWM gating signals. The current probe is LOTO C10B with a ±10 A input range. The conversion factor of the current probe is 2.5.

**Figure 10:**
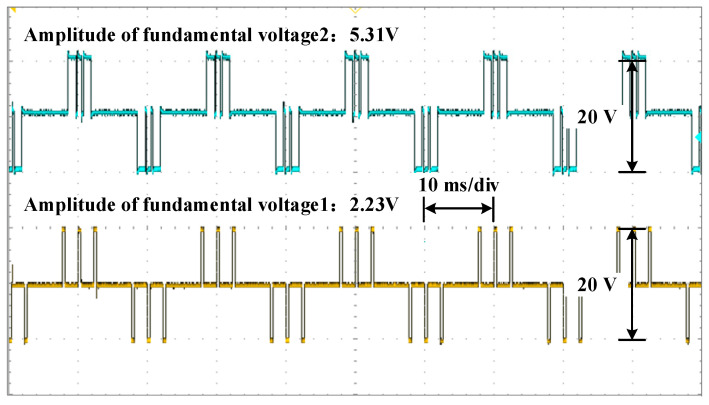
Voltage waveforms for each module at distribution ratio 0.3 with modulation index 0.3.

**Figure 11:**
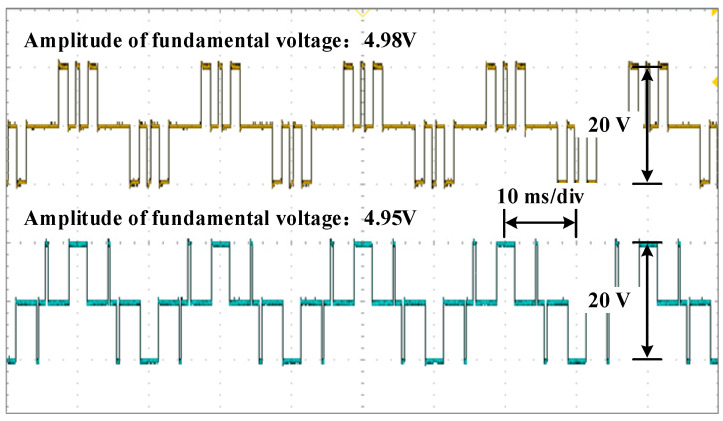
Voltage waveforms for each module at distribution ratio 0.5 with modulation index 0.4.

**Figure 12:**
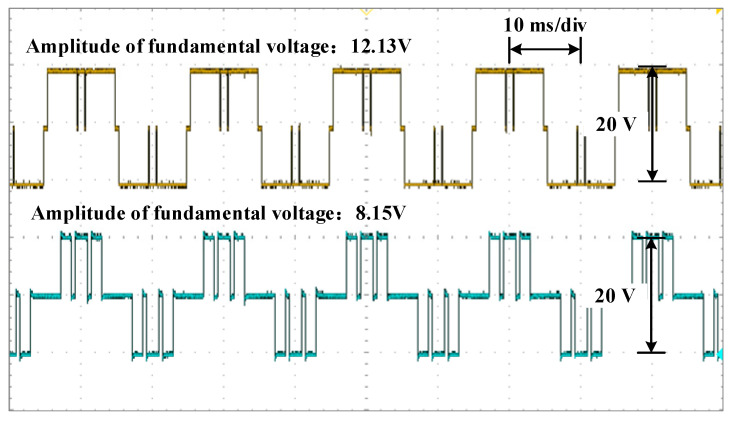
Voltage waveforms for each module at distribution ratio 0.6 with modulation index 0.8.

**Figure 13:**
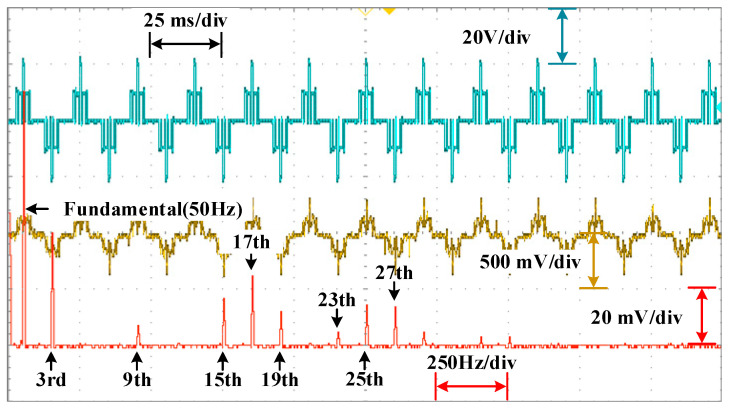
The voltage waveform, current waveform and FFT for the current at distribution ratio 0.3 with modulation index 0.3.

**Figure 14:**
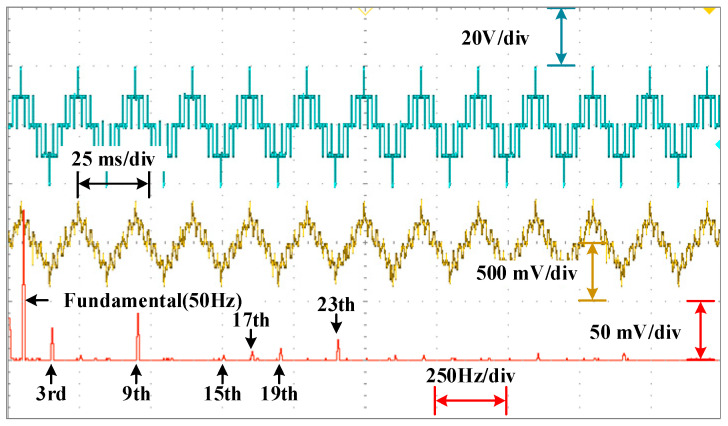
The voltage waveform, current waveform and FFT for the current at distribution ratio 0.5 with modulation index 0.4.

**Figure 15:**
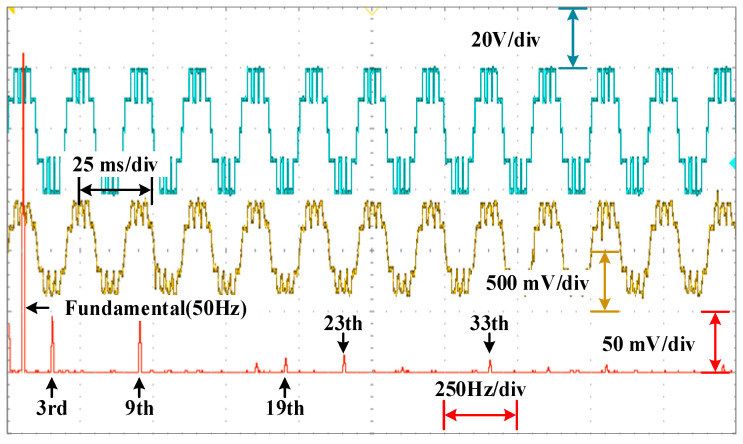
The voltage waveform, current waveform and FFT for the current at distribution ratio 0.6 with modulation index 0.8.

The waveforms of the voltage are shown in Figure 10, Figure 11 and Figure 12 for different modulation indexes and distribution ratios. Meanwhile, the voltage waveform, current waveform and fast Fourier transform (FFT) analysis of the load current are shown in Figure 13, Figure 14 and Figure 15.

As shown in Figure 13, Figure 14 and Figure 15, it can be seen that the low-frequency harmonics including 5th, 7th, 11th and 13th in the current have been perfectly eliminated, which verifies the effectiveness and superiority of the global optimal solutions for the PD–SHE strategy.

Based on Figure 1 and Figure 9, it is evident that the two modules are cascaded. Therefore, the currents through the two modules are identical. According to (4) and (5), whether the active power between modules is distributed proportionately depends on the ratio of the amplitude of the fundamental voltage. Therefore, it can be concluded that the active power of each module is distributed according to the distribution ratio in Figure 10, Figure 11 and Figure 12. Moreover, the active power of each module also can be calculated, and the results of theoretical and experimental values at different distribution ratios with modulation indexes are listed in Table 4.

As illustrated in Table 4, the experimental results show that the active powers of the two modules are almost perfectly distributed, as expected. Thus, the reliability of the global optimal solutions solved by the PHC algorithm in terms of power distribution is verified.

**Figure 16:**
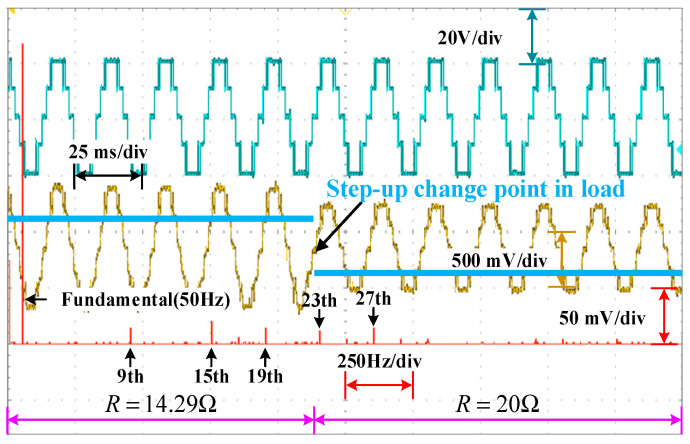
Dynamic behavior of system with step-up change in load.

**Figure 17:**
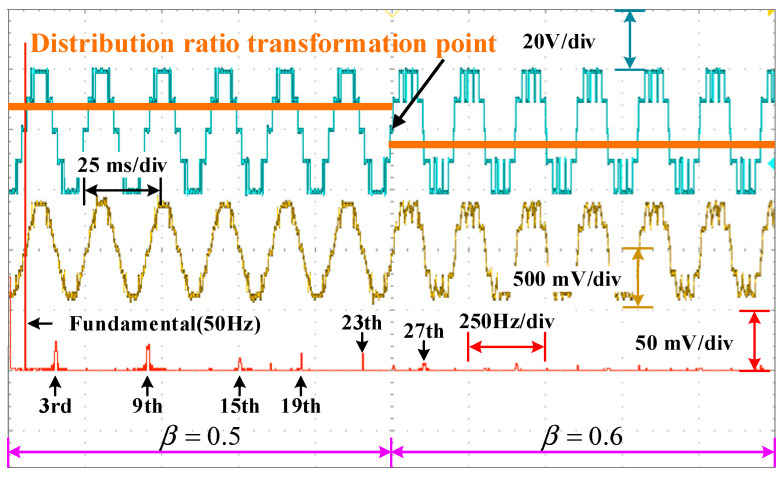
Dynamic behavior of system with transformation in distribution ratio.

Dynamic resistance and distribution ratio experiments are carried out to validate the dynamics of the system. In Figure 16, the resistance occurs a step-up change from 14.29 Ω to 20 Ω at the artificially setting switching point. During this period, abilities of the system to eliminate low-frequency harmonics and power distribution are still maintaining. In Figure 17, the distribution ratio is changed from 0.5 to 0.6 at the artificially setting switching point. Both the voltage and current waveforms switch smoothly. Moreover, the selective harmonics are eliminated perfectly and the active power is distributed expectedly. The change process shows that PD–SHE strategy has excellent dynamic characteristics. The changes in modulation index and power distribution ratios are achieved via the timer of STM32F407 without an ADC sensor, and the changes in load resistance are achieved by short-circuiting the series resistor.

**Figure 18:**
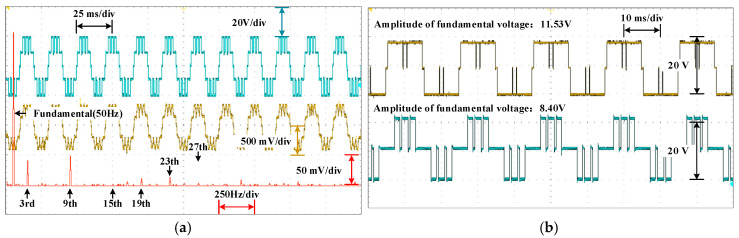
Behavior of system with unequal DC-link voltages. (**a**) The diagram of experimental waveform and its FFT analysis. (**b**) Comparison of power between two modules.

Experiment with DC-link voltages of 10.4 V and 9.5 V is conducted to verify the robustness of the PD–SHE, and its results are shown in Figure 18. From Figure 18, it can be seen that proposed PD–SHE has good robustness performance. These experiment results directly demonstrate that proposed PD–SHE has good robustness performance in terms of changes in the voltage levels, load resistance, and power distribution ratios.

Some strategies are taken into consideration in terms of switching frequency, carrier cycle and power distribution. The related results are listed in Table 5.

Table 5 shows the comparison of different strategies. It can be seen that the proposed PD–SHE strategy has advantages over those proposed in [12,23] in terms of switching frequency, carrier cycle, and power distribution. Additionally, in our experimental setup, the four gate-driving signals for the H-bridge inverter are generated by using global optimal theoretical solution and Output Compare Toggle mode of the STM32F407 timer module to guarantee experimental reproducibility.

## 7. Conclusions

In this article, a novel PD–SHE strategy is proposed. The PHC algorithm is utilized to solve the global optimal solutions of PD–SHE strategy which can distribute the active power arbitrarily for each H-bridge module while achieving the elimination of a selective low-frequency harmonic. Theoretically, the PD–SHE model is established by introducing a distribution ratio and the redundancy of solution is theoretically analyzed. Due to solid theoretical support, all the candidate theoretical solutions of PD–SHE equations can be found without the initial values by the PHC algorithm. Technically, the global optimal solutions can be selected by THD when there are multiple groups of candidate solutions for some modulation indexes. Moreover, the PD–SHE strategy can avoid the repeatability of the solutions by adding effective constraints. Compared with MPSO and CoDE algorithms, the PHC algorithm can not only obtain the global optimal solutions, but also avoid the risk of failure to solve the PD–SHE equations. Finally, in comparison with conventional power distributions method, experimental results verify that PD–SHE can achieve arbitrary power distribution and selective harmonic elimination but with a low switching frequency and a single carrier cycle. The limitation of the proposed method is that PD–SHE is solved offline but achieved online through a lookup table, which has a low execution efficiency and a large amount of data memory. Hence, future research should focus on exploring more effective online solving methods for PD–SHE strategy, such as some hybrid algorithms based on neural networks and numerical iterations.

## Figures and Tables

**Table 1:** The solutions for modulation index 0.8.

$\alpha_{k}$	Group 1	Group 2	Group 3
$\alpha_{1}$	3.90186°↑	5.06136°↑	**14.1741**°↑
$\alpha_{2}$	16.5816°↓	77.9622°↓	**20.4584**°↓
$\alpha_{3}$	40.4712°↑	89.2863°↑	**39.8817**°↑
$\alpha_{4}$	19.0567°↑	21.8144°↑	**22.1785**°↑
$\alpha_{5}$	64.5182°↓	64.1039°↓	**45.9325**°↓
$\alpha_{6}$	73.4359°↑	72.0403°↑	**55.2851**°↑
THD (%)	29.49	47.88	**19.97**

↑ represents the rising edge and ↓ represents the falling edge.

**Table 2:** Solutions for three algorithms with modulation index 0.8.

	β	β = 0.5			β = 0.6		
$\alpha_{k}$		MPSO [17]	CoDE [20]	PHC	MPSO [17]	CoDE [20]	PHC
$\alpha_{1}$		3.75543°↑	6.14530°↑	**14.1741**°↑	13.6401°↑	14.3065°↑	**4.81367**°↑
$\alpha_{2}$		6.92519°↓	64.2674°↓	**20.4584**°↓	32.7699°↓	19.1756°↓	**79.0750**°↓
$\alpha_{3}$		39.5251°↑	69.4066°↑	**39.8817**°↑	36.7221°↑	22.4359°↑	**81.1963**°↑
$\alpha_{4}$		15.9431°↑	37.2930°↑	**22.1785**°↑	34.8725°↑	42.6002°↑	**38.1153**°↑
$\alpha_{5}$		47.2100°↓	73.5535°↓	**45.9325**°↓	44.7971°↓	46.4249°↓	**64.6167**°↓
$\alpha_{6}$		54.6907°↑	77.9303°↑	**55.2851**°↑	55.8850°↑	56.4680°↑	**73.6262**°↑
TSHD (V)		0.33	0.25	**0**	0.65	0.24	**0**
AEAP (W)		0.55	1.26	**0**	0.39	0.67	**0**

↑ represents the rising edge and ↓ represents the falling edge.

**Table 3:** Parameters of the experimental platform.

Parameters	Values
DC-link Voltage 1 (V)	10
DC-link Voltage 2 (V)	10
Load Resistance (Ω)	20/14.29
Load Inductance (mH)	3.3
Fundamental Frequency (Hz)	50
Conversion Factor of the Current Probe	2.5

**Table 4:** Theoretical values versus experimental values.

M	β	Module	Theoretical Values				Experimental Values			
			$V_{f}$ (V)	$I_{L}$ (A)	P (W)	$P_{1}:P_{2}$	$V_{f}$ (V)	$I_{L}$ (A)	P (W)	$P_{1}:P_{2}$
0.3	0.3	Module 1	2.29	0.38	0.44	3:7	2.23	0.32	0.36	3:7.08
		Module 2	5.35		1.02		5.31		0.85	
0.4	0.5	Module 1	5.09	0.51	1.30	5:5	4.98	0.46	1.15	5:5
		Module 2	5.09		1.30		4.95		1.14	
0.8	0.6	Module 1	12.22	1.02	6.22	6:4	12.13	0.89	5.38	5.94:4
		Module 2	8.15		4.14		8.15		3.62	

**Table 5:** The comparative results of algorithms.

Strategy	Switching Frequency	Carrier Cycle	Power Distribution
RMM [12]	High	Multiple	Single
IPD–TPWM [23]	High	Multiple	Single
PD–SHE	Low	Single	Arbitrary

## Data Availability

The findings and original contributions of this study are detailed within the article. For additional information, please contact the corresponding author.
